# Validity and utility of self-reported weight gain since age 20 in a 30-year-old Japanese working population

**DOI:** 10.1093/joccuh/uiag052

**Published:** 2026-09-11

**Authors:** Satsue Nagahama, Ai Hori, Bibha Dhungel, Takehiro Michikawa, Keiko Asakura, Yuji Nishiwaki

**Affiliations:** All Japan Labour Welfare Foundation, Tokyo, Japan; Department of Environmental and Occupational Health, School of Medicine, Toho University, Tokyo, Japan; Department of Global Public Health, Institute of Medicine, University of Tsukuba, Tsukuba, Japan; Transborder Medical Center, Institute of Medicine, University of Tsukuba, Tsukuba, Japan; Melbourne School of Population and Global Health, University of Melbourne, Melbourne, Australia; Department of Environmental and Occupational Health, School of Medicine, Toho University, Tokyo, Japan; Department of Preventive Medicine, School of Medicine, Toho University, Tokyo, Japan; Department of Environmental and Occupational Health, School of Medicine, Toho University, Tokyo, Japan

**Keywords:** self-reported weight gain, cardiometabolic components, health checkup questionnaire, validity

## Abstract

**Objectives:**

We examined the validity of a questionnaire item on self-reported weight gain of ≥10 kg since age 20 among Japanese workers undergoing occupational health examinations. In addition, we performed the utility analysis to examine whether the associations with metabolic components at age 30 differ when using self-reported weight gain versus objectively measured weight gain as the exposure variable.

**Methods:**

Participants were 6272 men and 2055 women aged 30 with objectively measured body weight data at ages 20 and 30. Weight gain (≥10 kg vs <10 kg) was defined using both self-reported and objectively measured data. The validity of the questionnaire item on self-reported weight gain was assessed using objectively measured weight gain as the reference standard. To evaluate its utility, we used modified Poisson regression to examine the associations with cardiometabolic components at age 30, separately for men and women, and compared these associations between the two exposure definitions: self-reported and objectively measured weight gain.

**Results:**

At age 30, the sensitivity, specificity, and positive predictive value were 80.1%, 79.7%, and 52.9% in men, and 73.3%, 89.1%, and 48.5% in women, respectively. Associations of self-reported weight gain of ≥10 kg with cardiometabolic components at age 30 were similar in magnitude to those based on objectively measured weight gain.

**Conclusions:**

The self-reported questionnaire item showed acceptable validity and may serve as a practical alternative when historical body weight data are unavailable.

## Teaser

**Key points**
 

- **What is already known on this topic:** Weight gain since early adulthood is associated with increased metabolic risk. In many settings, historical weight records are unavailable, and self-reported weight gain is commonly used as a proxy, although its validity remains uncertain.
- **What this study adds:** Self-reported weight gain of ≥10 kg since age 20 showed similar associations with cardiometabolic diseases at age 30 compared with objectively measured weight gain. The validity of self-reported weight gain of ≥10 kg relative to measured weight change was also evaluated.
- **How this study might affect research, practice, or policy:** This questionnaire item may serve as a practical tool for identifying individuals at increased cardiometabolic risk in settings where historical weight data are unavailable, supporting its use in health screening and occupational health practice.

## 1. Introduction

Weight gain in adulthood is widely recognized as a major risk factor for cardiometabolic diseases, such as hypertension, type 2 diabetes and cardiovascular disease. Specifically, weight gain beginning in early adulthood (such as from age 20) has been associated with increased risk of adverse health outcomes.^1–8^ These prior studies have consistently demonstrated that excess weight gain from early adulthood is associated with elevated blood pressure, dyslipidemia, insulin resistance, and increased abdominal adiposity, all of which are key features of cardiometabolic risk. Given the recognized association between weight gain since age 20 and cardiometabolic risk, accurately assessing past weight is crucial for identifying individuals at risk. Measuring past weight is difficult, and often necessitates reliance on self-reported data. Various studies have shown that self-reported weight gain is susceptible to recall bias and reporting errors. For example, a Swedish study of individuals with a mean age of 71.6 years found systematic underestimation of weight recalled from 20 years prior—particularly among individuals with higher final body mass index (BMI) values and lower cognitive function.^9^ Meanwhile, among Japanese adults aged 34-61 years, a previous study reported that weight recalled from around age 25 was 1.3 kg higher than measured weight on average.^10^ Other studies, including analyses from NHANES^11^ and European cohorts,^12^ have also documented systematic errors in recalled body weight. These findings suggest that self-reported weight gain may differ from objectively measured weight change, raising the possibility of misclassification when questionnaire data are used. Therefore, validation of questionnaire-based assessment of weight gain is important in epidemiological research or clinical risk screening.

In Japan, the Ministry of Health, Labour and Welfare recommends the use of a standardized health questionnaire in Specific Health Checkups.^13^ Although Specific Health Checkups target individuals aged 40 years and older, the questionnaire itself is widely used across age groups in a variety of health screening settings, including occupational health checkups, community health checkups, and voluntary comprehensive health checkups. One of the items asks about self-reported weight gain: “Have you gained over 10 kg from your weight at age 20?” Because historical body weight measurements are often unavailable, this questionnaire item is widely used as a practical proxy for long-term weight gain in epidemiological studies and routine health screening practice.^7^^,^^8^^,^^14–16^ Several studies have reported associations between this questionnaire item and a variety of health outcomes, including chronic kidney disease, unhealthy lifestyle behaviors (eg, smoking, skipping breakfast, short sleep), and cardiometabolic risk factors.^7^^,^^8^ These findings suggest that the questionnaire item may be useful for identifying individuals at increased health risk. However, the validity of this self-reported weight gain measure has not been sufficiently established, raising the possibility that misclassification of weight gain status may have biased the observed associations with health outcomes. In addition, weight gain during early adulthood, particularly between ages 20 and 30, has been associated with an increased risk of future cardiometabolic diseases, including diabetes and hypertension.^17–19^ Therefore, establishing the validity of this questionnaire item for assessing early adult weight gain may facilitate early identification of high-risk individuals around age 30, before the age at which Specific Health Checkups begin.

Specifically, to the best of our knowledge, only a small number of studies have evaluated how well this questionnaire item reflects objectively measured weight gain.^20^^,^^21^ In addition, no previous studies have compared the associations between this questionnaire item and cardiometabolic components with those based on objectively measured weight gain. Therefore, evaluating both the validity and utility of this questionnaire item has important implications for public health practice and research, given that measured weight at age 20 is often unavailable in health screening and research settings.

This study aimed to evaluate the validity and utility of the self-reported questionnaire item, “Have you gained over 10 kg from your weight at age 20?” in a Japanese working-age population. The primary aim was to assess the validity of this questionnaire item by comparing self-reported weight gain with objectively measured weight change since age 20. The secondary aim was to compare the associations between self-reported weight gain (≥10 kg vs <10 kg) and cardiometabolic components at age 30 with those obtained using objectively measured weight gain (≥10 kg vs <10 kg) as the exposure variable.

## 2. Methods

### 2.1 Study design and participants

This study examined data from annual health checkups of Japanese workers conducted from fiscal years 2008 to 2023 by the All Japan Labour Welfare Association, which operates health checkup centers located in the prefectures of Aomori, Nagano, Yamagata, Ibaraki, Gunma, Tokyo, Kanagawa, and Aichi. Most participants underwent mandatory occupational health examinations conducted in accordance with the Industrial Safety and Health Act, while a small proportion underwent voluntary comprehensive health checkups or health checkups as dependents.

In the main analysis, the validity of the questionnaire was assessed in age-specific analyses (21-30 years) among participants who reached 20 years of age between fiscal years 2008 and 2013. Each analysis included participants with a body weight measurement at age 20 and both a questionnaire response and a body weight measurement at the corresponding target age (see Figure 1 for details). We performed cross-sectional analysis to assess the utility of the questionnaire for cardiometabolic components using health examination data from participants at age 30 between fiscal years 2018 and 2023. We present a flow chart of the study participants in Figure 1. First, we identified participants aged 20 between fiscal years 2008 and 2013 (*n* = 42 253; 18 381 women). Of the 42 253 participants, we excluded individuals who did not provide informed consent (*n* = 1), those with missing weight data (*n* = 265), and those who had no subsequent health checkup data after age 20 (*n* = 10 224), leaving 31 763 participants (18 475 men and 13 288 women). Additionally, when individuals underwent more than 1 health checkup within the same year, only 1 observation per individual per year was retained, prioritizing those with both measured weight and questionnaire responses; if multiple observations met these criteria, the earliest visit was selected. For the validity analyses, participants were included if they had a body weight measurement at age 20 and both a questionnaire response and a body weight measurement at the corresponding target age. Consequently, the number of participants varied across age-specific analyses, ranging from 23 526 at age 21 to 8327 at age 30. For the utility analyses, each cardiometabolic component was analyzed separately using available data, and the number of participants varied accordingly.

**Figure 1:**
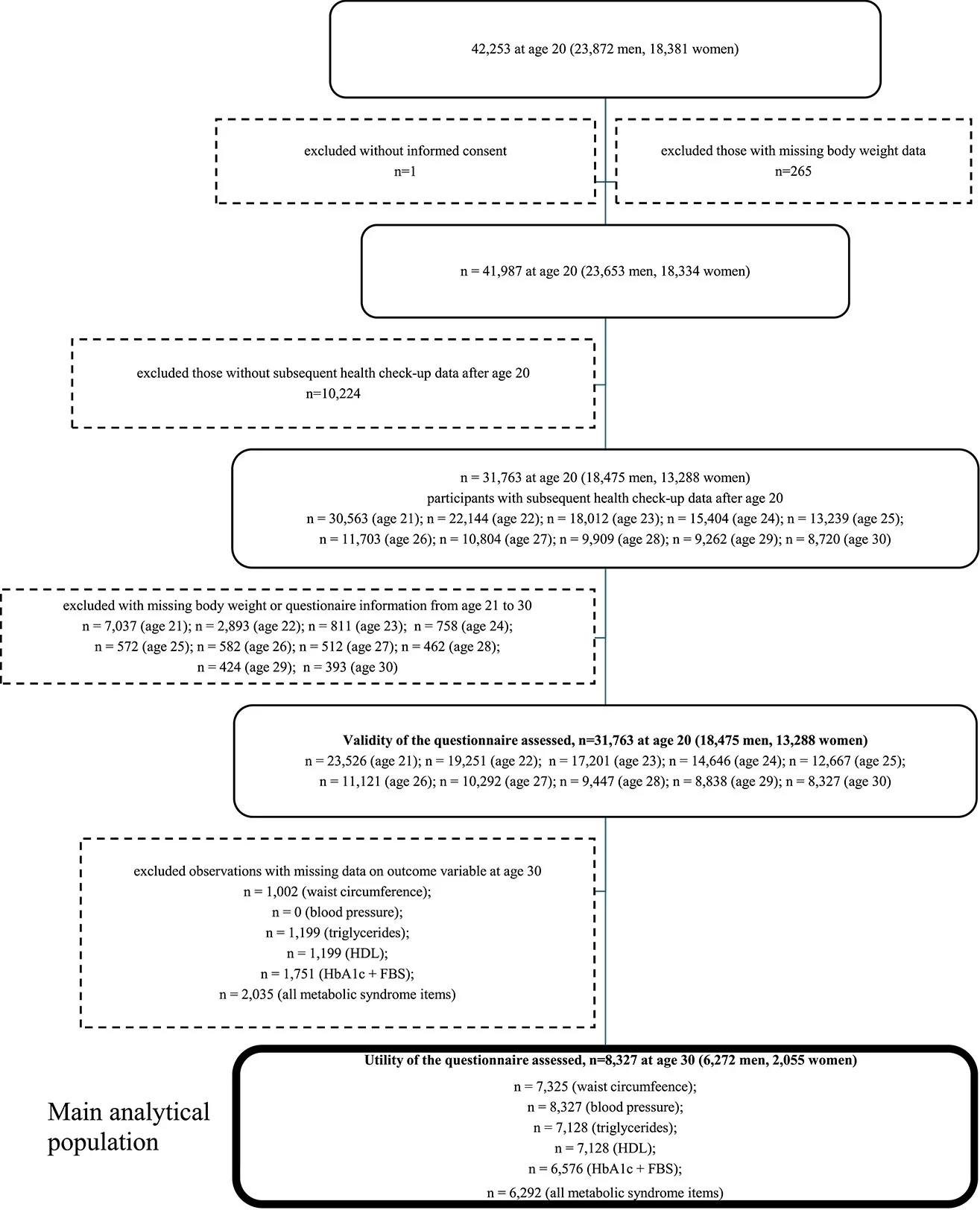
Flow chart of the study participants.

### 2.2 Cardiometabolic components assessment

Cardiometabolic components were defined according to the metabolic syndrome criteria of the National Cholesterol Education Program Adult Treatment Panel III (NCEP ATP III) (revised in 2004), with Asia-specific waist circumference thresholds.^22^ Participants were classified as having each cardiometabolic component as follows: waist circumference (≥90 cm for men, ≥80 cm for women); triglycerides (≥150 mg/dL or under treatment); high-density lipoprotein (HDL) cholesterol (<40 mg/dL for men, <50 mg/dL for women or under treatment); blood pressure (≥130/85 mmHg or under treatment); glucose metabolism (hemoglobin A1c [HbA1c] ≥6.0%, fasting blood glucose ≥100 mg/dL if available, or under treatment). Metabolic syndrome was defined as the presence of at least 3 of these components.

### 2.3 Statistical analyses

#### 2.3.1 General statistical methods

Baseline characteristics, including weight and BMI at ages 20 and 30, weight change from age 20 to 30, and cardiometabolic components at age 30 (systolic and diastolic blood pressure, HbA1c, HDL cholesterol, triglycerides, and waist circumference) were summarized as means and SDs. We also calculated the proportion of individuals at age 30 who met the criteria for each cardiometabolic component, overall metabolic syndrome prevalence, and the proportion receiving treatment for hypertension, diabetes, and dyslipidemia. In addition, we assessed lifestyle behaviors (smoking status, alcohol consumption, and physical activity), which were categorized as described below and used as adjustment variables.

Participants were classified using 2 primary variables. The first variable was objectively measured weight change from age 20 to 30, categorized as <10 kg or ≥10 kg. The second variable was based on self-reported responses to the question, “Have you gained over 10 kilograms from your weight at age 20?” with responses classified as “Yes” or “No.”^13^ Cross-tabulation of these 2 variables was used to classify participants into 4 groups according to whether the self-reported weight gain correctly reflected the measured weight gain (“correct” and “incorrect” in the tables, respectively). To assess potential selection bias, baseline characteristics at age 20 were compared between participants included in the analyses and those excluded from the analyses.

#### 2.3.2 Validity of the self-reported weight gain questionnaire

We evaluated the validity of the self-reported questionnaire item using sensitivity, specificity, area under the receiver operating characteristic curve, percentage correctly classified, positive and negative likelihood ratios, positive predictive value (PPV), and negative predictive value (NPV) for each year from age 21 to 30. Sensitivity was defined as the proportion of participants with ≥10 kg objectively measured weight gain since age 20 (eg, between ages 20 and 21 for 21-year-olds) who responded “Yes” on the questionnaire. Specificity was the proportion of participants with <10 kg objectively measured weight gain who responded “No.” These metrics were calculated separately for men and women. The proportions of participants classified as “correct” and “incorrect” based on questionnaire responses and objectively measured weight change were graphed for each year from age 21 to 30 separately for men and women.

#### 2.3.3 Utility of the self-reported weight gain questionnaire

Prevalence ratios (PRs) for cardiometabolic components and metabolic syndrome at age 30 were estimated using modified Poisson regression with 2 exposure definitions: objectively measured weight gain (<10 kg [reference] vs ≥10 kg) and responses to self-reported weight gain (“No” [reference] vs “Yes”).

Outcomes were systolic blood pressure (<130 mmHg vs ≥130 mmHg or under treatment), diastolic blood pressure (<85 mmHg vs ≥85 mmHg or under treatment), triglycerides (<150 mg/dL vs ≥150 mg/dL or under treatment), HDL cholesterol (≥40 mg/dL for men and ≥50 mg/dL for women vs <40 mg/dL for men and <50 mg/dL for women, or under treatment), glucose metabolism (HbA1c <6.0% and fasting blood glucose <100 mg/dL if available vs HbA1c ≥6.0% or fasting blood glucose ≥100 mg/dL if available, or under treatment), waist circumference (<90 cm for men and <80 cm for women vs ≥90 cm for men and ≥80 cm for women), and metabolic syndrome (absent vs present). Only participants with available data were included in each analysis. Model 1 presented unadjusted PRs; Model 2 was adjusted for smoking status (nonsmoker, ≤20 cigarettes/day, ≥21 cigarettes/day), physical activity (≥1 hour per day of walking or equivalent physical activity vs <1 hour per day), and alcohol consumption (nondrinker or rarely drinks, <1 *go*/day, 1-2 *go*/day, ≥2 *go*/day). A “*go*” is a traditional Japanese unit of volume, equivalent to approximately 180 mL, with 1 *go* containing approximately 23 g of pure alcohol. Model 3 was further adjusted for measured body weight at age 20.

### 2.4 Ethical considerations

This study was conducted in accordance with the principles outlined in the Helsinki Declaration. The research protocol was approved by the Ethics Committee of the Faculty of Medicine, Toho University (No. A24103_A21087_A20011_A18095_A17107_A16130). We obtained written informed consent from each participant who attended a heath checkup. Additionally, participants were provided with the option to opt-out, ensuring their autonomy and respect for their rights throughout the research process.

## 3. Results

Among participants eligible for the validity analysis, those included in the age 30 validity analysis had slightly higher body weight and BMI at age 20 than those excluded, whereas other characteristics at age 20 were generally similar (Table S1). Table 1 shows the characteristics of participants at age 30 included in the main analysis of metabolic outcomes, stratified by sex (6272 men and 2055 women). The average body weights at age 20 were 65.4 kg in men and 53.1 kg in women. Table S2A shows that among participants with ≥10 kg objectively measured weight gain, the average increase was similar in men and women (15.2 and 15.3 kg, respectively). Those with <10 kg gain exhibited smaller changes (2.4 kg in men and 1.1 kg in women). Table S2B shows that a higher proportion of participants self-reported ≥10 kg weight gain (2104 men [33.5%] and 382 women [18.6%]) than in the measured data (1389 men [22.1%] and 251 women [12.2%]). The prevalence of each cardiometabolic component was higher in those with ≥10 kg objectively measured weight gain than in those with self-reported ≥10 kg weight gain in both sexes. Table S3 presents participant characteristics according to self-reported and objectively measured weight gain (<10 kg or ≥10 kg). Among participants with <10 kg objectively measured weight gain, those who reported ≥10 kg had a higher average body weight at age 20 than those who reported <10 kg (71.7 vs 63.2 kg in men; 60.2 vs 51.6 kg in women). Conversely, participants with ≥10 kg objectively measured weight gain who self-reported <10 kg had a lower average body weight at age 20 than those who reported ≥10 kg (62.8 kg vs 68.5 kg in men; 55.2 kg vs 58.0 kg in women). In both sexes, a consistent gradient was observed in the prevalence of metabolic syndrome across the following groups (from lowest to highest): correctly reported <10 kg, incorrectly reported <10 kg (actual ≥10 kg), incorrectly reported ≥10 kg (actual <10 kg), and correctly reported ≥10 kg. The corresponding prevalences were 13.0%, 28.1%, 33.6%, and 49.2% in men, and 4.6%, 26.9%, 28.3%, and 33.8% in women, respectively.

**Table 1:** Characteristics of the participants.

Characteristics	Men	Women
**Number of participants, *n* (%)**	6272 (75)	2055 (25)
**Body weight at age 20, mean (SD), kg**	65.4 (13)	53.1 (10)
**Body weight at age 30, mean (SD), kg**	70.6 (15)	56.0 (12)
**Weight change from age 20 to age 30, mean (SD), kg**	5.2 (7.4)	2.9 (6.5)
**BMI at age 20, mean (SD), kg/m** ^**2**^	22.3 (4.1)	21.3 (3.7)
**BMI at age 30, mean (SD), kg/m** ^**2**^	24.0 (4.6)	22.3 (4.7)
**HbA1c at age 30, mean (SD), %**	5.3 (0.5)	5.3 (0.4)
**Systolic blood pressure at age 30, mean (SD), mmHg**	125 (14)	114 (14)
**Diastolic blood pressure at age 30, mean (SD), mmHg**	74 (11)	71 (10)
**Triglycerides at age 30, mean (SD), mg/dL**	123 (96)	80 (57)
**HDL cholesterol at age 30, mean (SD), mg/dL**	56 (14)	67 (15)
**Waist circumference at age 30, mean (SD), cm**	83.3 (12)	76.1 (11)
**Metabolic syndrome at age 30, *n* (%)**	1111 (23.6)	163 (10.3)
**Systolic blood pressure ≥130 mmHg at age 30, *n* (%)**	2230 (35.6)	249 (12.1)
**Diastolic blood pressure ≥85 mmHg at age 30, *n* (%)**	980 (15.6)	172 (8.4)
**Triglycerides ≥150 mg/dL at age 30, *n* (%)**	1343 (25.3)	149 (8.2)
**HDL cholesterol <40 mg/dL (men)/<50 mg/dL (women) at age 30, *n* (%)**	443 (8.3)	190 (10.5)
**HbA1c ≥6.0% or fasting blood glucose ≥100 mg/dL at age 30, *n* (%)**	260 (5.3)	41 (2.4)
**Waist circumference meeting NCEP-ATP III criteria at age 30, *n* (%)**	1407 (25.0)	488 (28.6)
**Smoking status at age 30, *n* (%)**		
** Nonsmoker**	3693 (59.0)	1850 (90.1)
** ≤20 cigarettes/day**	2259 (36.1)	200 (9.7)
** ≥21 cigarettes/day**	312 (5.0)	4 (0.2)
**Alcohol consumption at age 30,** ^**a**^ ***n* (%)**		
** Nondrinker or rarely drinks**	2598 (41.5)	1304 (63.5)
** <1 *go*/day**	2389 (38.1)	644 (31.4)
** 1–2 *go*/day**	1052 (16.8)	98 (4.8)
** ≥2 *go*/day**	228 (3.6)	8 (0.4)
**Daily physical activity ≥60 min/day, *n* (%)**	3366 (53.7)	671 (32.7)
**Self-reported weight gain ≥10 kg since age 20 (answering “Yes”),** ^**b**^ ***n* (%)**	2104 (33.6)	382 (18.6)
**Under treatment for hypertension at age 30, *n* (%)**	69 (1.1)	9 (0.4)
**Under treatment for diabetes at age 30, *n* (%)**	39 (0.6)	6 (0.3)
**Under treatment for dyslipidemia at age 30, *n* (%)**	42 (0.7)	5 (0.2)

Abbreviations: BMI: body mass index (kg/m^2^); HbA1c, hemoglobin A1c; HDL, high-density lipoprotein; NCEP-ATP III, National Cholesterol Education Program Adult Treatment Panel III.

^a^1 *go* ~180 mL sake, equivalent to approximately 23 g ethanol. ^b^Self-reported weight gain based on the questionnaire item asking: “Have you gained over 10 kg from your weight at age 20?” (response = Yes).

The proportion of participants who correctly responded to the self-reported weight gain item increased with age among those with ≥10 kg objectively measured weight gain (from 45.3% at age 21 to 80.1% at age 30 in men, and from 45.0% to 73.7% in women). In contrast, the proportion who correctly responded to the self-reported weight gain item decreased with age among those with <10 kg objectively measured weight gain (from 97.8% at age 21 to 79.7% at age 30 in men, and from 98.8% to 89.1% in women) (Figure 2). Sensitivity increased substantially from age 21 to 30, whereas specificity slightly decreased. At age 30, the area under the receiver operating characteristic curve was 0.80 in men and 0.81 in women, while PPV was 52.9% in men and 48.5% in women, and NPV was 93.4% and 96.1%, respectively (Table  S4).

**Figure 2:**
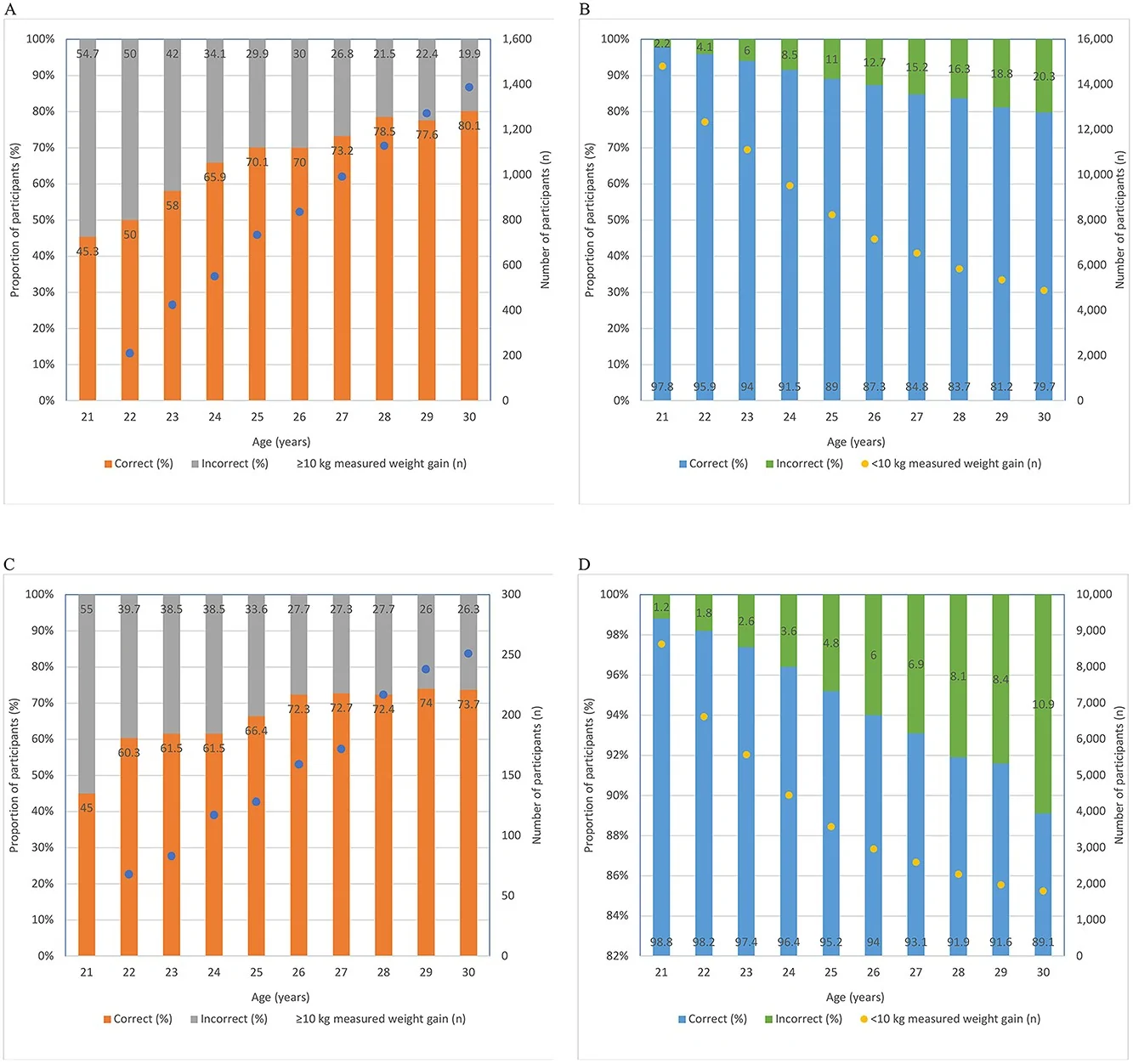
(A) Men with ≥10 kg objectively measured weight gain since age 20. Bars indicate the proportions of participants classified as “correct” and “incorrect” at each age among those with ≥10 kg objectively measured weight gain since age 20. “Correct” indicates participants who reported ≥10 kg weight gain, whereas “incorrect” indicates those who reported <10 kg weight gain. The number of these participants at each age is shown as blue dots (right y-axis). (B) Men with <10 kg objectively measured weight gain since age 20. Bars indicate the proportions of participants classified as “correct” and “incorrect” at each age among those with <10 kg objectively measured weight gain since age 20. “Correct” indicates participants who reported <10 kg weight gain, whereas “incorrect” indicates those who reported ≥10 kg weight gain. The number of these participants at each age is shown as yellow dots (right y-axis). (C) Women with ≥10 kg objectively measured weight gain since age 20. Bars indicate the proportions of participants classified as “correct” and “incorrect” at each age among those with ≥10 kg objectively measured weight gain since age 20. “Correct” indicates participants who reported ≥10 kg weight gain, whereas “incorrect” indicates those who reported <10 kg weight gain. The number of these participants at each age is shown as blue dots (right y-axis). (D). Women with <10 kg objectively measured weight gain since age 20. Bars indicate the proportions of participants classified as “correct” and “incorrect” at each age among those with <10 kg objectively measured weight gain since age 20. “Correct” indicates participants who reported <10 kg weight gain, whereas “incorrect” indicates those who reported ≥10 kg weight gain. The number of these participants at each age is shown as yellow dots (right y-axis).

Table 2 shows that the PRs were similar for objectively measured and self-reported weight gain using modified Poisson regression. In crude analyses (Model 1) and after adjustment for lifestyle factors (Model 2), PRs were comparable, with largely overlapping 95% CIs. After adjusting for body weight at age 20 (Model 3), results remained similar. In men, adjusted PRs for systolic blood pressure were 1.4 (95% CI, 1.3-1.5) for objectively measured weight gain ≥10 kg and 1.3 (95% CI, 1.2-1.4) for self-reported weight gain. Comparable PRs were observed for diastolic blood pressure (1.8 vs 1.6), triglycerides (2.1 vs 2.0), HDL cholesterol (2.8 vs 2.6), HbA1c and/or fasting blood glucose (2.0 vs 2.1), waist circumference (3.1 vs 3.2), and metabolic syndrome (2.4 vs 2.3). In women, PRs were generally comparable between objectively measured and self-reported weight gain, except that HbA1c and/or fasting blood glucose remained higher with objectively measured weight gain than with self-reported weight gain (2.9 vs 1.9).

**Table 2:** Prevalence ratios (with 95% CIs) for cardiometabolic components and metabolic syndrome at age 30 according to weight gain, estimated using modified Poisson regression.^a^

		Men					Women			
Variables	Exposure ^b,c^	Category	*n* (%)	Model 1	Model 2	Model 3	*n* (%)	Model 1	Model 2	Model 3
**SBP**	Measured	<10 kg	4883 (32.0)	ref.	ref.	ref.	1804 (9.8)	ref.	ref.	ref.
		≥10 kg	1389 (48.1)	1.5 (1.4–1.6)	1.5 (1.4–1.6)	1.4 (1.3–1.5)	251 (28.7)	2.9 (2.3–3.7)	2.9 (2.2–3.7)	2.2 (1.7–2.8)
	Self-reported	<10 kg	4168 (30.2)	ref.	ref.	ref.	1673 (9.0)	ref.	ref.	ref.
		≥10 kg	2104 (46.2)	1.5 (1.4–1.6)	1.5 (1.4-1.6)	1.3 (1.2–1.4)	382 (25.9)	2.9 (2.3–3.6)	2.9 (2.3-3.6)	1.9 (1.5–2.4)
**DBP**	Measured	<10 kg	4883 (13.1)	ref.	ref.	ref.	1804 (7.0)	ref.	ref.	ref.
		≥10 kg	1389 (24.6)	1.9 (1.7–2.1)	1.9 (1.7–2.1)	1.8 (1.6–2.0)	251 (18.3)	2.6 (1.9–3.6)	2.6 (1.9–3.6)	2.0 (1.4–2.7)
	Self-reported	<10 kg	4168 (11.8)	ref.	ref.	ref.	1673 (5.9)	ref.	ref.	ref.
		≥10 kg	2104 (23.3)	2.0 (1.8–2.2)	2.0 (1.8–2.2)	1.6 (1.4–1.8)	382 (19.1)	3.2 (2.4–4.3)	3.2 (2.4–4.3)	2.1 (1.5–2.8)
**TG**	Measured	<10 kg	4137 (19.8)	ref.	ref.	ref.	1591 (5.9)	ref.	ref.	ref.
		≥10 kg	1174 (44.7)	2.3 (2.1–2.5)	2.2 (2.1–2.5)	2.1 (2.0–2.3)	226 (24.8)	4.2 (3.1–5.7)	4.2 (3.1–5.7)	3.6 (2.6–5.0)
	Self-reported	<10 kg	3508 (17.9)	ref.	ref.	ref.	1482 (4.9)	ref.	ref.	ref.
		≥10 kg	1803 (39.7)	2.2 (2.0–2.4)	2.2 (2.0-2.4)	2.0 (1.8–2.2)	335 (22.7)	4.6 (3.4–6.2)	4.6 (3.4–6.2)	3.8 (2.8–5.3)
**HDL-C**	Measured	<10 kg	4137 (77.9)	ref.	ref.	ref.	1591 (87.6)	ref.	ref.	ref.
		≥10 kg	1174 (22.1)	3.1 (2.6–3.6)	3.0 (2.5–3.6)	2.8 (2.4–3.3)	226 (12.4)	3.2 (2.4–4.2)	3.1 (2.4–4.1)	2.6 (2.0–3.4)
	Self-reported	<10 kg	3508 (66.1)	ref.	ref.	ref.	1482 (81.6)	ref.	ref.	ref.
		≥10 kg	1803 (33.9)	3.1 (2.6-3.8)	3.1 (2.6–3.7)	2.6 (2.2–3.2)	335 (18.4)	3.4 (2.6–4.4)	3.3 (2.6–4.3)	2.6 (1.9–3.4)
**HbA1c**	Measured	<10 kg	3774 (4.2)	ref.	ref.	ref.	1488 (1.8)	ref.	ref.	ref.
		≥10 kg	1095 (9.2)	2.2 (1.7–2.8)	2.2 (1.7–2.8)	2.0 (1.6–2.5)	219 (6.8)	3.9 (2.1–7.3)	3.9 (2.1–7.2)	2.9 (1.6–5.3)
	Self-reported	<10 kg	3203 (3.3)	ref.	ref.	ref.	1387 (1.6)	ref.	ref.	ref.
		≥10 kg	1666 (9.2)	2.7 (2.2–3.5)	2.7 (2.1–3.5)	2.1 (1.6–2.6)	320 (5.9)	3.7 (2.1–6.8)	3.7 (2.0–6.9)	1.9 (1.0–3.9)
**WC**	Measured	<10 kg	4362 (16.2)	ref.	ref.	ref.	1492 (20.8)	ref.	ref.	ref.
		≥10 kg	1259 (55.6)	3.4 (3.2–3.7)	3.4 (3.1–3.7)	3.1 (2.8–3.4)	212 (84.0)	4.0 (3.6–4.5)	4.0 (3.6–4.5)	3.1 (2.7–3.6)
	Self-reported	<10 kg	3704 (11.4)	ref.	ref.	ref.	1389 (17.4)	ref.	ref.	ref.
		≥10 kg	1917 (51.3)	4.5 (4.1–5.0)	4.5 (4.1–4.9)	3.2 (2.9–3.5)	315 (78.1)	4.5 (3.9–5.1)	4.5 (4.0–5.1)	3.1 (2.7-3.6)
**MetS** ^**d**^	Measured	<10 kg	3651 (17.3)	ref.	ref.	ref.	1376 (7.2)	ref.	ref.	ref.
		≥10 kg	1065 (45.2)	2.6 (2.4–2.9)	2.6 (2.4–2.9)	2.4 (2.2–2.7)	200 (32.0)	4.4 (3.4–5.9)	4.4 (3.4–5.9)	3.4 (2.6–4.6)
	Self-reported	<10 kg	3097 (14.0)	ref.	ref.	ref.	1276 (5.5)	ref.	ref.	ref.
		≥10 kg	1619 (41.9)	3.0 (2.7–3.3)	2.9 (2.7–3.3)	2.3 (2.0–2.5)	300 (31.0)	5.7 (4.3–7.5)	5.6 (4.2–7.6)	3.6 (2.6–4.9)

Abbreviations: DBP, diastolic blood pressure; HbA1c, hemoglobin A1c; HDL-C, high-density lipoprotein cholesterol; MetS, metabolic syndrome; NCEP-ATP III, National Cholesterol Education Program Adult Treatment Panel III; SBP, systolic blood pressure; TG, triglycerides; WC, waist circumference.

^a^Prevalence ratios were estimated using modified Poisson regression. Model 1: crude model. Model 2: adjusted for smoking, alcohol drinking, and exercise habits. Model 3: further adjusted for body weight at age 20. ^b^Measured: objectively measured weight gain since age 20. ^c^Self-reported: self-reported weight gain since age 20 based on the questionnaire item. ^d^MetS was defined according to the modified NCEP-ATP III criteria (2005) with Asia-specific waist circumference thresholds: ≥90 cm in men and ≥80 cm in women; triglycerides ≥150 mg/dL or under treatment; HDL-C <40 mg/dL in men or <50 mg/dL in women or under treatment; blood pressure ≥130/85 mmHg or under treatment; and glucose metabolism defined as HbA1c ≥6.0%, fasting blood glucose ≥100 mg/dL (if available), or under treatment. MetS was defined as the presence of ≥3 of these components.

## 4. Discussion

This study demonstrated the validity and utility of a self-reported questionnaire on weight gain since age 20. Validity improved from age 21 to 30, reaching an acceptable level by age 30 in terms of sensitivity and specificity. At age 30, self-reported weight gain of ≥10 kg showed similar associations with cardiometabolic components to those based on objectively measured weight gain. These findings suggest that this widely used questionnaire item is a practical indicator for identifying individuals at risk of cardiometabolic diseases related to long-term weight gain in health guidance and epidemiological research, particularly when objectively measured weight at age 20 is unavailable.

### 4.1 Validity of the questionnaire and comparison with prior studies

The questionnaire demonstrated sensitivity and specificity of approximately 80% at age 30, supporting its use in population-based risk screening. Sensitivity increased substantially from age 21 to 30, while specificity showed a slight decline, suggesting that awareness of weight gain may improve over time, although it may be accompanied by a modest increase in overreporting. Although this specific questionnaire item has not been directly evaluated in previous studies, existing literature on recall bias in self-reported weight provides useful context for interpreting our findings. Previous studies have reported that individuals with higher body weight tend to underreport their current or past weight.^23–25^ In contrast, our findings showed a modest level of overreporting, with more participants self-reporting ≥10 kg weight gain than was objectively measured. The discrepancy may partly reflect differences in question framing. Earlier studies required participants to recall their actual body weight at age 20, whereas our questionnaire asked only whether their current weight was ≥10 kg higher than at age 20. These differences should be taken into account when interpreting self-reported weight data in population-based assessments.

### 4.2 Utility of self-reported weight gain

Both self-reported and objectively measured weight gain ≥10 kg showed similar associations with cardiometabolic components at age 30. Adjustment for lifestyle factors and objectively measured weight at age 20 did not materially change these associations, suggesting little influence of body size in early adulthood. Several previous studies of Japanese populations examined the associations between weight gain since the age of 20 and adverse health outcomes using the same questionnaire item, indicating the widespread use of this item in clinical and epidemiological settings. These studies have reported similar associations to ours, including associations with chronic kidney disease,^8^^,^^16^ nonalcoholic fatty liver disease,^15^ and metabolic abnormalities.^7^^,^^26^ Kaneko et al^26^ reported hazard ratios for metabolic outcomes in a middle-aged population (mean age 44.7 years). These estimates were somewhat lower than those observed in our younger population, suggesting that weight gain earlier in life may confer a higher relative metabolic risk, consistent with previous reports highlighting early adulthood (20-30 years) as a critical period for future cardiometabolic risk.^17–19^ These findings indicate that this questionnaire performs similarly to objectively measured weight gain in epidemiological analyses of cardiometabolic outcomes.

### 4.3 Interpretation and implications of the questionnaire

Our findings suggest that this questionnaire may serve as a useful screening tool for identifying individuals at increased cardiometabolic risk and may also be used as a surrogate for objectively measured weight gain since age 20 in epidemiological research. However, discrepancies between self-reported and measured weight gain status remain clinically relevant. The prevalence of cardiometabolic diseases at age 30 was highest among participants with ≥10 kg objectively measured weight gain who correctly self-reported it, followed by those with <10 kg objectively measured weight gain who incorrectly self-reported ≥10 kg, those with ≥10 kg objectively measured weight gain who incorrectly self-reported <10 kg, and those with <10 kg objectively measured weight gain who correctly self-reported it, in both men and women. Self-reported weight gain may capture aspects of cardiometabolic risk not fully reflected by objective measures. Importantly, those who incorrectly self-reported <10 kg despite objectively measured weight gain ≥10 kg had limited awareness of their weight gain and may be at risk of overlooked health issues if the questionnaire is used alone. Previous studies in middle-aged and older adults have reported that weight reduction during midlife is associated with reduced health risks.^27^ Although our study population was younger, intervention among participants with weight gain may reduce health risks. Overall, while caution is needed for individuals with limited awareness of their weight gain, this questionnaire may still be useful for identifying individuals at elevated cardiometabolic risk when historical weight data are unavailable.

### 4.4 Strengths and limitations

The current study had several strengths. First, it included both self-reported and objectively measured body weight data at ages 20 and 30, allowing us to assess the validity of self-reported weight gain and compare their associations with cardiometabolic outcomes across the 2 exposure definitions. Second, standardized health checkup measurements conducted by trained staff helped ensure the reliability of the body weight measurements. Third, the questionnaire used to assess self-reported weight gain was derived from the standardized national health checkup questionnaire developed by the Japanese Ministry of Health, Labour and Welfare, which has been widely used nationwide since 2008. This enhances the practical relevance of our findings for health screening practice in Japan.

However, several limitations of this study should be noted. Body weight at age 20 was based on a single measurement within a 1-year period, which may not have captured short-term fluctuations around the time of the health check-up. Consequently, self-reported weight gain may have been affected by the timing of the health checkup. In addition, our primary outcomes were limited to metabolic health indicators assessed at age 30, while many metabolic abnormalities develop later in life. Longer-term follow-up beyond age 30 is warranted. Furthermore, although selection bias due to participant exclusion cannot be ruled out, baseline characteristics were generally similar between included and excluded participants, suggesting a limited impact on our findings. Finally, this study was conducted among an occupational population in Japan, which may limit the generalizability of the findings to other populations or nonworking individuals.

## 5. Conclusions

The validity of the Japanese Ministry of Health, Labour and Welfare–recommended questionnaire item on self-reported weight gain of ≥10 kg since age 20 was supported by comparisons with objectively measured weight change since age 20. Self-reported weight gain on this questionnaire item also showed similar associations with cardiometabolic abnormalities at age 30 to those based on objectively measured weight gain. These findings suggest that in epidemiological research this widely used questionnaire item may serve as a practical tool for identifying individuals at elevated risk of cardiometabolic abnormalities and as a surrogate for objectively measured weight gain since age 20 when historical measured weight data are unavailable.

## Supplementary Material

Sup_file_20260819_uiag052

## Data Availability

The data are not publicly available due to ethical considerations but are available from the corresponding author on reasonable request, subject to approval by the relevant ethics committee.
