# Supplementary material for: Validity and utility of self-reported weight gain since age 20 in a 30-year-old Japanese working population
Source: J Occup Health. 2026 Sep 11;68(1):uiag052. doi: 10.1093/joccuh/uiag052 (PMC13690000; doi:10.1093/joccuh/uiag052)
Supplement: Supplementary file 1 [file sup_file_20260819_uiag052.docx]

| Supplementary Table S1. Characteristics of included and excluded participants (n=31,763) | | | | |  | | |
| --- | --- | --- | --- | --- | --- | --- | --- |
|  | Men | |  | Women | | | |
|  | Included | Excluded |  | Included | | Excluded | |
| Number of participants, n (%) | 6,272 (34) | 12,203 (66) |  | 2,055 (15) | | 11,233 (85) | |
| Body weight at age 20, mean (SD), kg | 65.4 (13) | 65.1 (13) |  | 53.1 (10.0) | | 52.5 (9.0) | |
| BMI at age 20, mean (SD), kg/m² | 22.3 (4.1) | 22.2 (4.0) |  | 21.3 (3.7) | | 21.0 (3.3) | |
| Systolic blood pressure at age 20, mean (SD), mmHg | 123 (13) | 122 (13) |  | 112 (12) | | 111 (12) | |
| Diastolic blood pressure at age 20, mean (SD), mmHg | 68 (9.1) | 68 (9.3) |  | 65 (9.2) | | 65 (8.9) | |
| Triglycerides at age 20, mean (SD), mg/dL | 95.6 (66) | 97.5 (67) |  | 70.0 (41) | | 71.9 (47) | |
| HDL cholesterol at age 20, mean (SD), mg/dL | 56.2 (12) | 56.2 (12) |  | 65.3 (12) | | 66.0 (13) | |
| Waist circumference at age 20, mean (SD), cm | 77.0 (11) | 77.0 (11) |  | 72.1 (9.2) | | 72.6 (8.3) | |
| Systolic blood pressure ≥130 mmHg at age 20, % | 29.7 | 30.2 |  | 7.5 | | 7.1 | |
| Diastolic blood pressure ≥85 mmHg at age 20, % | 3.5 | 4.2 |  | 2.8 | | 2.3 | |
| Triglycerides ≥150 mg/dL at age 20, % | 13.8 | 15 |  | 4.3 | | 5.1 | |
| HDL cholesterol <40 mg/dL (men) / <50 mg/dL (women) at age 20, % | 5.0 | 4.7 |  | 8.6 | | 8.5 | |
| Waist circumference meeting NCEP-ATP III criteria at age 20, % | 12.2 | 11.5 |  | 16.8 | | 14.9 | |
| Smoking status at age 20, % |  |  |  |  | |  | |
| Non-smoker | 57.0 | 54.2 |  | 86.8 | | 85.8 | |
| ≤20 cigarettes/day | 38.4 | 40.5 |  | 12.7 | | 13.3 | |
| ≥21 cigarettes/day | 4.6 | 5.4 |  | 0.50 | | 0.82 | |
| Alcohol consumption at age 20, % |  |  |  |  | |  | |
| Non-drinker or rarely drinks | 44.6 | 42.1 |  | 55.4 | | 54.6 | |
| <1 go/day | 44.2 | 45.4 |  | 40.6 | | 39.7 | |
| 1–2 go/day | 10.5 | 11.6 |  | 4.0 | | 5.4 | |
| ≥2 go/day | 0.7 | 0.92 |  | 0.0 | | 0.25 | |
| Daily physical activity ≥60 min/day at age 20, % | 29.7 | 31.6 |  | 19.7 | | 21.9 | |
| Under treatment for hypertension at age 20, % | 0.05 | 0.07 |  | 0.05 | | 0.04 | |
| Under treatment for diabetes at age 20, % | 0.10 | 0.09 |  | 0.05 | | 0.07 | |
| Under treatment for dyslipidaemia at age 20, % | 0.06 | 0.02 |  | 0.15 | | | 0.01 |
| Included participants were those included in the validity analysis . Excluded participants were those excluded from the validity analysis because of missing data required for the analysis. BMI: body mass index (kg/m²). 1 go ≈ 180 mL sake, equivalent to approximately 23 g ethanol. NCEP-ATP III: National Cholesterol Education Program Adult Treatment Panel III. Values are presented for participants with available data for each variable. Therefore, the number of participants may vary across variables because of missing data. | | | | | | | |

| Supplementary Table S2-A. Participant Characteristics by Objectively Measured Weight Gain | | | | | |
| --- | --- | --- | --- | --- | --- |
|  | Men | |  | Women | |
|  | ＜10㎏ | ≥10㎏ |  | ＜10㎏ | ≥10㎏ |
| Number of participants, n (%) | 4,883 (78) | 1,389 (22) |  | 1,804 (88) | 251 (12) |
| Body weight at age 20, mean (SD), kg | 64.9 (13) | 67.3 (13) |  | 52.5 (9.6) | 57.2 (12) |
| Body weight at age 30, mean (SD), kg | 67.3 (13) | 82.5 (15) |  | 53.7 (9.9) | 72.5 (14) |
| Weight change from age 20 to age 30, mean (SD), kg | 2.4 (5.1) | 15.2 (5.0) |  | 1.1 (4.4) | 15.3 (5.4) |
| BMI at age 20, mean (SD), kg/m² | 22.2 (4.1) | 22.7 (4.1) |  | 21.1 (3.6) | 22.8 (4.4) |
| BMI at age 30, mean (SD), kg/m² | 22.9 (4.0) | 27.7 (4.8) |  | 21.4 (3.8) | 28.8 (5.4) |
| HbA1c at age 30, mean (SD), % | 5.3 (0.5) | 5.4 (0.5) |  | 5.2 (0.4) | 5.4 (0.4) |
| Systolic blood pressure at age 30, mean (SD), mmHg | 123 (14) | 130 (14) |  | 113 (14) | 123 (15) |
| Diastolic blood pressure at age 30, mean (SD), mmHg | 73 (10) | 78 (11) |  | 70 (10) | 76 (10) |
| Triglycerides at age 30, mean (SD), mg/dL | 109 (83) | 169 (120) |  | 74 (50) | 116 (83) |
| HDL cholesterol at age 30, mean (SD), mg/dL | 58 (14) | 50 (11) |  | 68 (14) | 59 (14) |
| Waist circumference at age 30, mean (SD), cm | 80.4 (10) | 93.5 (12) |  | 74.0 (9.1) | 91.1 (12) |
| Metabolic syndrome at age 30, % | 17.3 | 45.2 |  | 7.2 | 32.0 |
| Systolic blood pressure ≥130 mmHg at age 30, % | 32.0 | 48.1 |  | 9.8 | 28.7 |
| Diastolic blood pressure ≥85 mmHg at age 30, % | 13.1 | 24.6 |  | 7.0 | 18.3 |
| Triglycerides ≥150 mg/dL at age 30, % | 19.8 | 44.7 |  | 5.9 | 24.8 |
| HDL cholesterol <40 mg/dL (men) / <50 mg/dL (women) at age 30, % | 5.7 | 17.6 |  | 8.2 | 26.1 |
| HbA1c ≥6.0% or fasting blood glucose ≥100 mg/dL at age 30, % | 4.2 | 9.2 |  | 1.8 | 6.9 |
| Waist circumference meeting NCEP-ATP III criteria at age 30, % | 16.2 | 55.6 |  | 20.8 | 84.0 |
| Smoking status at age 30, % |  |  |  |  |  |
| Non-smoker | 59.2 | 58.2 |  | 90.0 | 90.4 |
| ≤20 cigarettes/day | 36.2 | 35.7 |  | 9.8 | 9.6 |
| ≥21 cigarettes/day | 4.7 | 6.1 |  | 0.22 | 0.00 |
| Alcohol consumption at age 30, % |  |  |  |  |  |
| Non-drinker or rarely drinks | 41.4 | 41.7 |  | 63.3 | 64.8 |
| <1 go/day | 38.5 | 36.9 |  | 31.7 | 29.2 |
| 1–2 go/day | 16.7 | 17.2 |  | 4.7 | 5.6 |
| ≥2 go/day | 3.5 | 4.3 |  | 0.4 | 0.40 |
| Daily physical activity ≥60 min/day, % | 54.0 | 52.7 |  | 32.4 | 34.7 |
| Self-reported weight gain ≥10 kg since age 20 (answering “Yes”), % | 20.3 | 80.1 |  | 10.9 | 73.7 |
| Under treatment for hypertension at age 30, % | 1.0 | 1.4 |  | 0.28 | 1.60 |
| Under treatment for diabetes at age 30, % | 0.7 | 0.5 |  | 0.22 | 0.80 |
| Under treatment for dyslipidaemia at age 30, % | 0.6 | 0.9 |  | 0.11 | 1.2 |
| BMI: body mass index (kg/m²). 1 go ≈ 180 mL sake, equivalent to approximately 23 g ethanol. NCEP-ATP III: National Cholesterol Education Program Adult Treatment Panel III. Self-reported weight gain based on the questionnaire item asking: “Have you gained over 10 kg from your weight at age 20?” (response = Yes). | | | | | |

| Supplementary Table S2-B. Participant Characteristics by Self-Reported Weight Gain at Age 30 | | | | | |
| --- | --- | --- | --- | --- | --- |
|  | Men | |  | Women | |
|  | ＜10㎏ | ≥10㎏ |  | ＜10㎏ | ≥10㎏ |
| Number of participants, n (%) | 4,168 (67) | 2,104 (34) |  | 1,673 (81) | 382 (19) |
| Body weight at age 20, mean (SD), kg | 63.1 (12) | 70.0 (14) |  | 51.7 (9.0) | 59.1 (12) |
| Body weight at age 30, mean (SD), kg | 65.6 (12) | 80.7 (15) |  | 52.8 (9.4) | 69.6 (14) |
| Weight change from age 20 to age 30, mean (SD), kg | 2.4 (5.6) | 10.8 (7.3) |  | 1.1 (4.8) | 10.5 (7.3) |
| BMI at age 20, mean (SD), kg/m² | 21.6 (3.8) | 23.7 (4.3) |  | 20.7 (3.3) | 23.7 (4.5) |
| BMI at age 30, mean (SD), kg/m² | 22.3 (3.7) | 27.2 (4.6) |  | 21.1 (3.5) | 27.8 (5.2) |
| HbA1c at age 30, mean (SD), % | 5.3 (0.5) | 5.4 (0.5) |  | 5.2 (0.3) | 5.4 (0.6) |
| Systolic blood pressure at age 30, mean (SD), mmHg | 123 (14) | 130 (14) |  | 112 (14) | 121 (15) |
| Diastolic blood pressure at age 30, mean (SD), mmHg | 73 (10) | 78 (11) |  | 70 (10) | 76 (11) |
| Triglycerides at age 30, mean (SD), mg/dL | 105 (79) | 157 (114) |  | 73 (46) | 111 (83) |
| HDL cholesterol at age 30, mean (SD), mg/dL | 59 (14) | 51 (11) |  | 69 (14) | 59 (14) |
| Waist circumference at age 30, mean (SD), cm | 78.9 (9.6) | 92.0 (11) |  | 73.2 (8.6) | 88.7 (12) |
| Metabolic syndrome at age 30, % | 14.0 | 41.9 |  | 5.5 | 31.0 |
| Systolic blood pressure ≥130 mmHg at age 30, % | 30.2 | 46.2 |  | 9.0 | 25.9 |
| Diastolic blood pressure ≥85 mmHg at age 30, % | 11.8 | 23.3 |  | 5.9 | 19.1 |
| Triglycerides ≥150 mg/dL at age 30, % | 17.9 | 39.7 |  | 4.9 | 22.7 |
| HDL cholesterol <40 mg/dL (men) / <50 mg/dL (women) at age 30, % | 4.9 | 15.1 |  | 7.3 | 24.5 |
| HbA1c ≥6.0% or fasting blood glucose ≥100 mg/dL at age 30, % | 3.3 | 9.2 |  | 1.6 | 5.9 |
| Waist circumference meeting NCEP-ATP III criteria at age 30, % | 11.4 | 51.3 |  | 17.4 | 78.1 |
| Smoking status at age 30, % |  |  |  |  |  |
| Non-smoker | 59.2 | 58.4 |  | 90.4 | 88.5 |
| ≤20 cigarettes/day | 36.7 | 34.8 |  | 9.5 | 10.7 |
| ≥21 cigarettes/day | 4.1 | 6.8 |  | 0.06 | 0.8 |
| Alcohol consumption at age 30, % |  |  |  |  |  |
| Non-drinker or rarely drinks | 41.6 | 41.1 |  | 63.5 | 63.3 |
| <1 go/day | 38.7 | 36.9 |  | 31.6 | 30.5 |
| 1–2 go/day | 16.4 | 17.6 |  | 4.6 | 5.5 |
| ≥2 go/day | 3.3 | 4.4 |  | 0.30 | 0.79 |
| Daily physical activity ≥60 min/day, % | 54.7 | 51.8 |  | 32.9 | 31.4 |
| Self-reported weight gain ≥10 kg since age 20 (answering “Yes”), % | 0 | 100 |  | 0 | 100 |
| Under treatment for hypertension at age 30, % | 0.8 | 1.6 |  | 0.2 | 1.6 |
| Under treatment for diabetes at age 30, % | 0.6 | 0.8 |  | 0.1 | 1.1 |
| Under treatment for dyslipidaemia at age 30, % | 0.5 | 1.1 |  | 0.2 | 0.5 |
| BMI: body mass index (kg/m²). 1 go ≈ 180 mL sake, equivalent to approximately 23 g ethanol. NCEP-ATP III: National Cholesterol Education Program Adult Treatment Panel III. Self-reported weight gain based on the questionnaire item asking: “Have you gained over 10 kg from your weight at age 20?” (response = Yes). | | | | | |

| Supplementary Table 3. Characteristics of Participants (age 30) | | | | | | | | | |
| --- | --- | --- | --- | --- | --- | --- | --- | --- | --- |
|  | Men | | | |  | Women | | | |
| Objectively Measured Weight Gain | <10㎏ | | ≥10㎏ | |  | ＜10㎏ | | ≥10㎏ | |
| Self-reported Weight Gain | <10kg | ≥10㎏ | <10kg | ≥10㎏ |  | <10kg | ≥10㎏ | <10kg | ≥10㎏ |
|  | correct | incorrect | incorrect | correct |  | correct | incorrect | incorrect | correct |
| Number of participants, n (%) | 3,891 (80) | 992 (20) | 277 (20) | 1,112 (80) |  | 1,607 (89) | 197 (11) | 66 (26) | 185 (74) |
| Body weight at age 20, mean (SD), kg | 63.2 (12) | 71.7 (14) | 62.8 (12) | 68.5 (12.8) |  | 51.6 (8.9) | 60.2 (12) | 55.2 (12) | 58.0 (11) |
| Body weight at age 30, mean (SD), kg | 64.9 (11) | 76.8 (13) | 75.5 (13) | 84.2 (15) |  | 52.2 (8.7) | 65.3 (12) | 67.9 (13) | 74.2 (14) |
| Weight change from age 20 to age 30, mean (SD), kg | 1.7 (5.1) | 5.1 (4.5) | 12.7 (2.8) | 15.8 (5.3) |  | 0.63 (4.2) | 5.2 (4.0) | 12.7 (3.4) | 16.2 (5.6) |
| BMI at age 20, mean (SD), kg/m² | 21.6 (3.9) | 24.5 (4.5) | 21.3 (3.8) | 23.1 (4.1) |  | 20.7 (3.2) | 24.2 (4.7) | 22.1 (4.7) | 23.1 (4.3) |
| BMI at age 30, mean (SD), kg/m² | 22.1 (3.5) | 26.1 (4.0) | 25.4 (4.2) | 28.3 (4.8) |  | 20.8 (3.2) | 26.3 (4.5) | 27.2 (5.3) | 29.4 (5.4) |
| HbA1c at age 30, mean (SD), % | 5.3 (0.5) | 5.4 (0.6) | 5.3 (0.3) | 5.4 (0.5) |  | 5.2 (0.3) | 5.4 (0.7) | 5.4 (0.4) | 5.4 (0.4) |
| Systolic blood pressure at age 30, mean (SD), mmHg | 123 (14) | 128 (13) | 127 (14) | 131 (14) |  | 112 (13) | 119 (15) | 122 (16) | 123 (14) |
| Diastolic blood pressure at age 30, mean (SD), mmHg | 73 (10) | 76 (10) | 76 (10) | 79 (11) |  | 69 (10) | 74 (11) | 75 (11) | 77 (10) |
| Triglycerides at age 30, mean (SD), mg/dL | 103 (77) | 136 (98) | 144 (95) | 175 (125) |  | 71 (45) | 101 (77) | 103 (63) | 121 (89) |
| Waist circumference at age 30, mean (SD), cm | 78.3 (9.2) | 88.8 (10) | 87.8 (11) | 94.8 (12) |  | 72.7 (8.0) | 85.1 (10) | 87.9 (9.6) | 92.1 (12) |
| Metabolic syndrome at age 30, % | 13.0 | 33.6 | 28.1 | 49.2 |  | 4.6 | 28.3 | 26.9 | 33.8 |
| Systolic blood pressure ≥130 mmHg at age 30, % | 29.6 | 41.4 | 38.6 | 50.5 |  | 8.2 | 22.8 | 27.3 | 29.2 |
| Diastolic blood pressure ≥85 mmHg at age 30, % | 11.2 | 20.3 | 19.1 | 26.0 |  | 5.6 | 18.3 | 13.6 | 20.0 |
| Triglycerides ≥150 mg/dL at age 30, % | 16.6 | 32.2 | 37.8 | 46.3 |  | 4.4 | 18.2 | 18.0 | 27.3 |
| HDL cholesterol <40 mg/dL (men) / <50 mg/dL (women) at age 30, % | 4.4 | 10.8 | 11.3 | 19.0 |  | 6.8 | 20.0 | 18.0 | 29.1 |
| HbA1c ≥6.0% or fasting blood glucose ≥100 mg/dL at age 30, % | 3.4 | 7.4 | 2.9 | 10.7 |  | 1.1 | 6.8 | 11.3 | 5.1 |
| Waist circumference meeting NCEP-ATP III criteria at age 30, % | 10.0 | 40.1 | 31.7 | 61.2 |  | 15.1 | 69.2 | 75.5 | 86.8 |
| Smoking status at age 30, % |  |  |  |  |  |  |  |  |  |
| Non-smoker | 59.3 | 58.8 | 58.5 | 58.2 |  | 90.5 | 85.8 | 87.9 | 91.4 |
| ≤20 cigarettes/day | 36.6 | 34.5 | 38.3 | 35.1 |  | 9.4 | 12.7 | 12.1 | 8.7 |
| ≥21 cigarettes/day | 4.2 | 6.8 | 3.3 | 6.8 |  | 0.06 | 1.5 | 0.00 | 0.00 |
| Alcohol consumption at age 30, % |  |  |  |  |  |  |  |  |  |
| Non-drinker or rarely drinks | 41.4 | 41.2 | 44.4 | 41.0 |  | 63.2 | 64.5 | 72.7 | 62.0 |
| <1 go/day | 39.0 | 36.5 | 35.4 | 37.3 |  | 31.9 | 30.0 | 24.2 | 31.0 |
| 1–2 go/day | 16.4 | 17.6 | 15.5 | 17.6 |  | 4.7 | 4.6 | 3.0 | 6.5 |
| ≥2 go/day | 3.2 | 4.6 | 4.7 | 4.1 |  | 0.3 | 1.0 | 0.00 | 0.5 |
| Daily physical activity ≥60 min/day, % | 54.5 | 52.1 | 57.8 | 51.4 |  | 32.8 | 28.9 | 36.4 | 34.1 |
| Under treatment for hypertension at age 30, % | 0.85 | 1.6 | 0.72 | 1.6 |  | 0.19 | 1.0 | 0.00 | 2.2 |
| Under treatment for diabetes at age 30, % | 0.57 | 1.0 | 0.36 | 0.54 |  | 0.06 | 1.5 | 1.5 | 0.54 |
| Under treatment for dyslipidaemia at age 30, % | 0.49 | 1.0 | 0.00 | 1.2 |  | 0.12 | 0.00 | 1.5 | 1.1 |
| “Self-reported weight gain was assessed using the questionnaire item: “Have you gained over 10 kg from your weight at age 20?”  “Correct/Incorrect” indicates whether the self-reported response was consistent with the objectively measured weight change. | | | | | | | | | |

| Supplementary Table S4. Sensitivity, Specificity, and Related Validity Measures of the Questionnaire Item | | | | | | | | | | |
| --- | --- | --- | --- | --- | --- | --- | --- | --- | --- | --- |
| Age, year | 21 | 22 | 23 | 24 | 25 | 26 | 27 | 28 | 29 | 30 |
| Men, n | 14,874 | 12,558 | 11,542 | 10,079 | 8,960 | 7,998 | 7,526 | 6,966 | 6,629 | 6,272 |
| AUC | 0.72 (0.65-0.78) | 0.73 (0.70-0.76) | 0.76 (0.74-0.78) | 0.79 (0.77-0.81) | 0.80 (0.78-0.81) | 0.79 (0.77-0.80) | 0.79 (0.78-0.80) | 0.81 (0.80-0.82) | 0.79 (0.78-0.81) | 0.80 (0.79-0.81) |
| Sensitivity | 45.3 | 50.0 | 58.0 | 65.9 | 70.1 | 70.0 | 73.2 | 78.5 | 77.6 | 80.1 |
| Specificity | 97.8 | 95.9 | 94.0 | 91.5 | 89.0 | 87.3 | 84.8 | 83.7 | 81.2 | 79.7 |
| Classified | 97.5 | 95.2 | 92.7 | 90.1 | 87.4 | 85.5 | 83.3 | 82.8 | 80.5 | 79.8 |
| LR+ | 20.2 | 12.3 | 9.7 | 7.6 | 6.4 | 5.5 | 4.8 | 4.8 | 4.1 | 3.9 |
| LR- | 0.56 | 0.52 | 0.45 | 0.37 | 0.34 | 0.34 | 0.32 | 0.26 | 0.28 | 0.25 |
| PPV, % | 8.2 | 17.2 | 26.9 | 31.0 | 36.2 | 39.1 | 42.2 | 48.2 | 49.5 | 52.9 |
| NPV, % | 99.8 | 99.1 | 98.3 | 97.9 | 97.1 | 96.1 | 95.4 | 95.3 | 93.8 | 93.4 |
| Women, n | 8,652 | 6,693 | 5,659 | 4,567 | 3,707 | 3,123 | 2,766 | 2,481 | 2,209 | 2,055 |
| AUC | 0.72 (0.60-0.83) | 0.79 (0.73-0.85) | 0.79 (0.74-0.85) | 0.80 (0.74-0.83) | 0.81 (0.77-0.85) | 0.83 (0.80-0.87) | 0.83 (0.80-0.86) | 0.82 (0.80-0.85) | 0.83 (0.80-0.86) | 0.81 (0.79-0.84) |
| Sensitivity | 45.0 | 60.3 | 61.5 | 61.5 | 66.4 | 72.3 | 72.7 | 72.4 | 74.0 | 73.7 |
| Specificity | 98.8 | 98.2 | 97.4 | 96.4 | 95.2 | 94.0 | 93.1 | 91.9 | 91.6 | 89.1 |
| Classified | 98.7 | 97.9 | 96.9 | 95.5 | 94.2 | 92.9 | 91.8 | 90.2 | 89.7 | 87.2 |
| LR+ | 37.4 | 34.1 | 24.0 | 17.1 | 13.9 | 12.0 | 10.5 | 9.0 | 8.8 | 6.7 |
| LR- | 0.56 | 0.40 | 0.40 | 0.40 | 0.35 | 0.29 | 0.29 | 0.30 | 0.28 | 0.30 |
| PPV, % | 8.0 | 25.6 | 26.0 | 31.0 | 33.1 | 39.3 | 41.1 | 46.1 | 51.5 | 48.5 |
| NPV, % | 99.9 | 99.6 | 99.4 | 99.0 | 98.8 | 98.4 | 98.1 | 97.2 | 96.7 | 96.1 |
